# Electron Spin–Lattice Relaxation of Substitutional Nitrogen in Silicon: The Role of Disorder and Motional Effects

**DOI:** 10.3390/nano14010021

**Published:** 2023-12-20

**Authors:** Matteo Belli, Marco Fanciulli

**Affiliations:** 1 CNR-IMM, Unità di Agrate Brianza, Via C. Olivetti, 2, 20864 Agrate Brianza, Italy; 2 Dipartimento di Scienza dei Materiali, Università degli Studi di Milano-Bicocca, Via R. Cozzi 55, 20125 Milano, Italy

**Keywords:** deep donors in silicon, nitrogen donor, electron paramagnetic resonance, spin–lattice relaxation, stretched exponential relaxation, isotopic purification, Avogadro project

## Abstract

In a previous investigation, the authors proposed nitrogen as a possible candidate for exploiting the donor spin in silicon quantum devices. This system is characterized by a ground state deeper than the other group V impurities in silicon, offering less stringent requirements on the device temperature necessary to access the unionized state. The nitrogen donor is slightly displaced from the substitutional site, and upon heating, the system undergoes a motional transition. In the present article, we show the results from our investigation on the spin–relaxation times in ^nat^Si and ^28^Si substrates and discuss the motional effects on relaxation. The stretched exponential relaxation observed is interpreted as a distribution of spin–lattice relaxation times, whose origin is also discussed. This information greatly contributes to the assessment of a nitrogen-doped silicon system as a potential candidate for quantum devices working at temperatures higher than those required for other group V donors in silicon.

## 1. Introduction

The electron and nuclear spins of donors in silicon represent a viable implementation of qubit systems for future quantum devices [1,2,3,4]. Often, standard donors, such as phosphorus or arsenic, are used [5,6,7,8], although other systems, such as bismuth [9,10,11,12] or double donors (Te and Se) [3,13,14], have been investigated to exploit a larger Hilbert space or different donor ionization regimes, respectively. Substitutional nitrogen (N_Si_) in silicon, a further example of the former case, with its ground state estimated at 336 meV below the bottom of the Si conduction band [15], has been recently investigated theoretically as a candidate system for room temperature single electron tunneling devices [16]. The depth of the donor ground state extends the freeze-out regime of the donor to relatively high temperatures. This hints at possible advantages for the substitutional–nitrogen system in terms of spin coherence or the thermal range of operation of quantum-based devices.

The N_Si_ center, termed SL5, has also been largely investigated in the past because of its unusual nature among the family of silicon donors. Its ground state is characterized by an [111] off-center configuration, which implies the presence of dynamical reorientations at room temperature [17,18,19,20,21,22,23,24,25,26,27,28,29,30]. Electron paramagnetic resonance (EPR) is naturally suited for the investigation of N_Si_ due to its paramagnetic ground state; furthermore, it can follow the center thermal evolution due to the time scale of EPR experiments.

In the present article, we use pulse EPR to probe the spin–lattice relaxation in the SL5 center and to determine how it is affected by the system’s complex dynamics. In particular, we compare ^28^Si:N (sample A) [31] and ^nat^Si:N (sample B), with similar donor concentrations. We observe a peculiar thermal evolution of stretched exponential spin–lattice relaxation, which is interpreted in the framework of a distribution of relaxation rates. A resulting thermal evolution of the spin–lattice relaxation rates for the most represented spin packets can be inferred and can be interpreted as due to direct and Raman mechanisms with exponents three and five, as often observed in Jahn–Teller systems.

## 2. Materials and Methods

The present investigation compares two nitrogen-doped silicon systems, one characterized by ^28^Si isotopic enrichment (sample A) and one by the natural abundance of Si isotopes (sample B). The ^28^Si isotopically enriched sample was derived from the bulk ^28^Si piece classified as “Si28-10-Pr11 part 5B4.2.2.1c (100)” and kindly provided by the Avogadro project consortium [31]. The sample was polished to reduce the intensity of the EPR cut signal in silicon and then irradiated with ^14^N^+^ ions with the following parameters: dose 5·10^15^ at/cm^2^, beam energy 190 keV, and tilting angle 7°. The implanted nitrogen donors were partially activated at substitutional sites by irradiation with ruby laser pulses with power J = 3.2 J/cm^2^ in various 3 mm × 3 mm square spots covering the whole implanted area. The natural abundance of undoped silicon reference samples underwent the same ^14^N^+^ irradiation and laser activation. A sketch of the preparation procedures can be found in Supplementary Discussion, Section S1.

The donor concentration was expected to follow the typical implantation profile; nevertheless, an average nitrogen concentration could be defined, and it could be indirectly measured via the method of instantaneous diffusion [5,6,9]. In the samples under investigation, we estimated the average substitutional nitrogen concentration to be comparable; they were determined to be (1.4 ± 0.3) × 10^18^ cm^−3^ and (1.8 ± 0.3) × 10^18^ cm^−3^ for sample A and sample B, respectively.

Electron paramagnetic resonance experiments were performed via a Bruker Elexsys E580 instrument equipped with a continuous flow cryostat and a cylindrical cavity (ER4188X-MD5) suited for X-band continuous-wave (cw) and pulsed investigations. Pulsed investigations for the measurement of spin–relaxation rates were performed on the high-field isolated line along the <111> orientation. Above the motional transition, the same orientation was maintained, following the displacement of the transition towards the averaged position. The length of the 90° pulses was set to 28 ns to obtain uniform irradiation of the transition while reducing the irradiation of the other transitions in the spectrum to a minimum. The width of the boxcar integrator window was set accordingly for the same purpose, with different parameters for the investigated samples due to the different line widths in the two cases. The spin–lattice relaxation times were measured via inversion recovery pulse sequences with echo detection, while spin–spin relaxation times were monitored by varying the interpulse delay in echo decay measurements, as described in the results section. In the case of inversion recovery measurements, the experimental traces were generally measured at least up to 15 times the value of T_1_ (obtained by a preliminary fit to a single exponential recovery) to improve a further fit with a stretched exponential recovery functional form. This was not possible at low temperatures due to a built-in limitation in the instrument control software. The code developed for the fitting of the angular variations made extensive use of EASYSPIN routines [32]. The uncertainty in the temperature values was generally kept lower than 0.2 K. In the temperature range approximately coinciding with the onset of motional averaging of the EPR spectrum, the experimental intensity decreased considerably, causing longer measurement times and larger experimental uncertainties.

## 3. Results

### 3.1. SL5 Center Angular Variation

**Figure 1:**
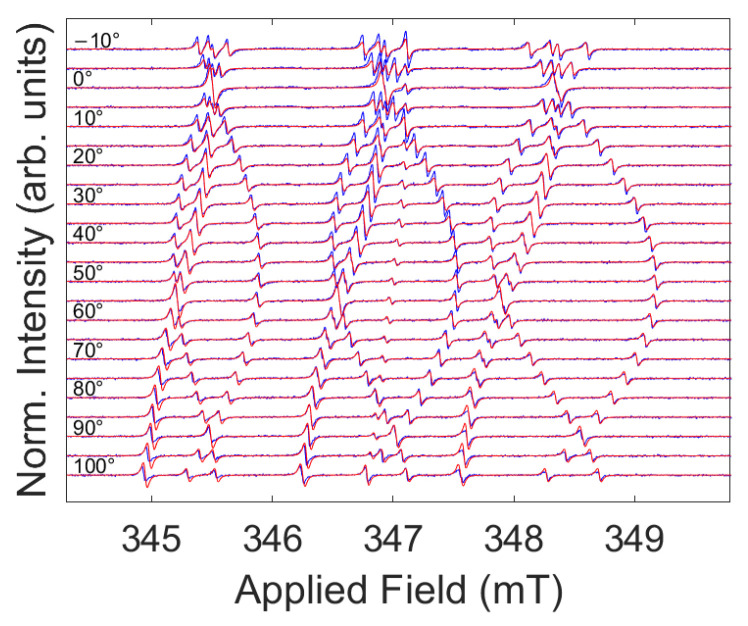
Angular variation in the electron paramagnetic resonance spectrum for sample A recorded at 80 K. The applied microwave power and modulation amplitude were 20 µW and 5 µT, respectively. Blue traces indicate experimental data, and red traces indicate the best fit according to Hamiltonian parameters as reported in Supplementary Table S1.

The EPR analyses on both samples found that the SL5 centers, i.e., the N_Si_ defects, were the main contributors to the spectrum [1,25,26,27,28,29]. In Figure 1, we show the low temperature angular variation in the continuous-wave (cw) spectra for sample A as an example, with its corresponding best fit (red lines). In the Supplementary Discussion, Section S2, we show the full set of angular variation continuous-wave EPR data for both samples as figures and tables and report the best fit parameters (Supplementary Table S2). In Supplementary Discussion, Section S3, we also include cw EPR saturation data for sample A (Supplementary Figure S5).

### 3.2. Spin–Relaxation Rates

**Figure 2:**
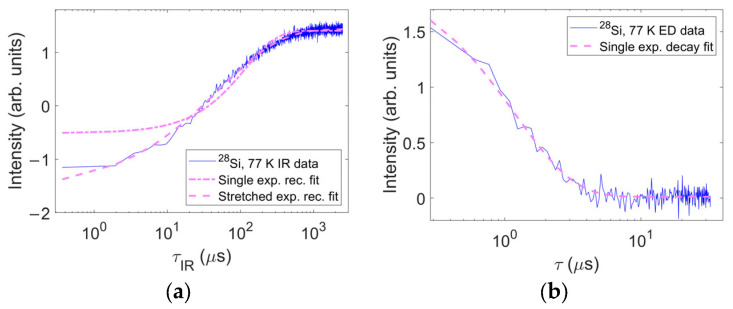
Typical examples of pulsed sequences for sample A, ^28^Si:N. (**a**) Inversion recovery data measured at 77 K (blue trace) with the corresponding single exponential recovery fit (pale purple dash–dotted trace) and stretched exponential recovery fit (pale purple dashed trace); (**b**) echo decay signal measured at 77 K (blue trace) with its single exponential decay fit (pale purple dashed trace). It is evident that while the echo decay can be interpreted as a single exponential relaxation, a single exponential recovery function does not grasp the details of the inversion recovery measurements. This is true in various temperature ranges.

The spin–relaxation rates were generally measured by applying standard pulse sequences. The spin–lattice relaxation was measured with the inversion recovery sequence and echo detection: 180°–$\tau_{IR}$–90°–τ–180°–τ–acquisition, where $\tau_{IR}$ indicates the inversion recovery delay and t is the echo interpulse delay. The alternative method of shot repetition time saturation for sample B was used only at the lowest temperatures as a test to reduce the acquisition times. This method consisted of monitoring the echo intensity as a function of the measurement repetition time. The spin–spin relaxation was monitored by measuring the echo decay, i.e., by varying the interpulse delay in an echo sequence (90°–τ–180°–τ–acquisition). Figure 2 shows examples of typical microwave experiments (other examples, complemented by graphs of fit residuals, are found in the Supplementary Discussion, Section S4).

The echo decay measurements could be effectively fitted by single exponential decays for both samples in the entire investigated temperature range (4.5 K–RT), as follows:

(1) $$I=I_{0}\cdot e^{-\frac{2\tau }{T_{2}}},$$

where I represents the experimental intensity, $I_{0}$ is the decay amplitude, and $T_{2}$ is the spin–spin relaxation time.

However, a single exponential recovery was not sufficient for the fitting of spin–lattice relaxation measurements; in certain temperature ranges, it was unsuccessful in fitting the features of the experimental curve. These temperature ranges nearly coincided for the two samples, indicating a possible common, unknown mechanism.

Hence, a different fitting model was applied. Since no other paramagnetic centers overlapped the isolated high-field transition along the <111> direction, the biexponential recovery model was discarded in favor of a stretched exponential model, expressed by the following equation:

(2) $$I=I_{0}\cdot \left(1-\left(2-\alpha \right)\cdot e^{-{\left(\frac{\tau_{IR}}{T_{1,Str}}\right)}^{n}}\right),$$

where I represents the measured intensity, $I_{0}$ describes the amplitude of the recovery, $T_{1,Str}$ is the stretched spin–lattice relaxation time, n is the exponent held within the [0, 1] range inherent in the stretched relaxation, and $\alpha =e^{-{\left(\frac{SRT-\tau_{IR}}{T_{1,Str}}\right)}^{n}}$ is a correction factor due to the finite shot repetition time (SRT). This correction factor is needed because, as an instrumental limitation, only an experiment frequency (${SRT}^{-1}$) could be set instead of a set delay between the experiments. This implies that when $\tau_{IR}$ is comparable to the shot repetition time, the delay between consecutive experiments is reduced with respect to the case of lower $\tau_{IR}$ values; thus, the correct theoretical functional form for the recovery tends to approach saturation recovery rather than inversion recovery.

**Figure 3:**
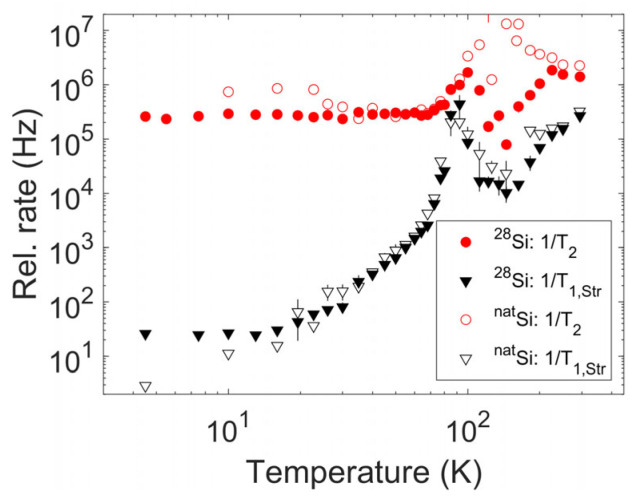
Spin–spin and stretched spin–lattice relaxation times for both investigated samples. Uncertainties on the relaxation rates as obtained from the fit of inversion recovery and echo decay data are shown as vertical bars for each experimental point or falls within the marker size.

**Figure 4:**
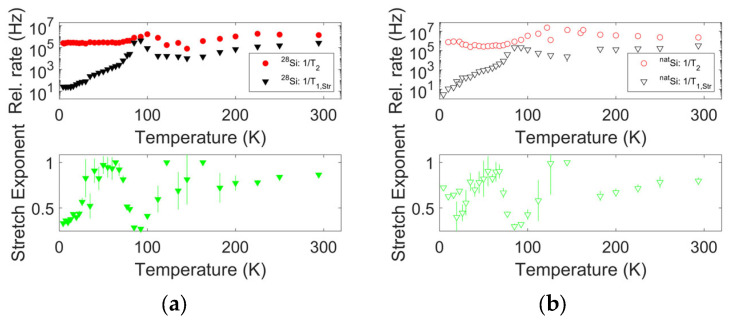
Spin–relaxation data as shown in Figure 3, separated for the two samples (upper panels). (**a**) sample A; (**b**) sample B. The lower panels show the corresponding stretched relaxation exponent. Uncertainties on the relaxation rates and the stretch exponent as obtained from the fit of inversion recovery and echo decay data are shown as vertical bars for each experimental point or falls within the marker size.

The resulting temperature trend for the spin–spin and spin–lattice relaxation times extracted according to the previous equation can be found in Figure 3 and Figure 4; in the lower panels of Figure 4, the $T_{1,Str}$ and $T_{2}$ trends can be compared with the stretch exponent values in the different temperature ranges.

**Figure 5:**
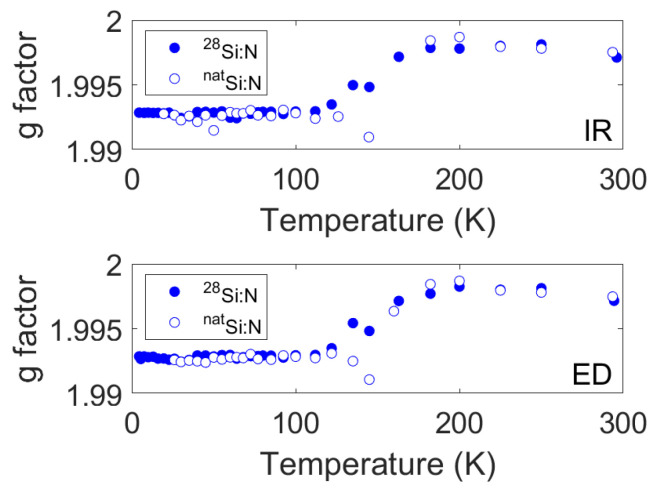
The g factor scale values correspond to the center of the transition. Such a g factor was selected for spin–relaxation measurements (upper panel for inversion recovery experiments, lower panel for echo decay experiments). The shift of the resonance follows motional averaging due to the motional transition of the SL5 center.

The $T_{1,Str}$ values nearly overlap for the two samples. The same observation occurs for most $T_{2}$ values, although in the range of the motional transition and above, some slight discrepancies emerge. These discrepancies disappear only at room temperature. Notably, the reported values were recorded by following the transition center across the motional transition, as reported in Figure 5.

### 3.3. Spin–Spin Relaxation

Spin–spin relaxation is essentially never limited by spin–lattice relaxation in the investigated temperature range. It roughly reaches a constant value, presumably determined by the concentration of SL5 centers in the two samples. By heating above approximately 80 K, the trend of $T_{2}$ values shows a peak earlier than the onset of the motional transition; afterward, the peak shifts towards the room temperature value. This peak is narrow in the isotopically enriched sample and broad in the naturally abundant sample, widening towards the high temperature range. An average of the $T_{2}$ values at temperatures lower than 80 K yields approximately 3–4 µs. The intrinsic spin–spin relaxation should be slightly longer since the reported spin–spin relaxation is affected by the instantaneous diffusion (ID) effect, i.e., a dipolar contribution to the relaxation rate due to the pulsed measurement method used to measure the relaxation itself. This is expressed by equation $\frac{1}{T_{2}}=\frac{1}{T_{2,0}}+\frac{1}{T_{ID}}$, where $\frac{1}{T_{2,0}}$ is the intrinsic spin–spin relaxation rate and $\frac{1}{T_{ID}}$ is the instantaneous diffusion rate. This effect is, however, rather small; for example, at 80 K, the intrinsic spin–spin relaxation time calculated by taking ID into account in the case of sample A is 3.1(1) µs. The experimental line width fitted from the cw spectra based on Lorentzian line shapes $\frac{1}{T_{2}}=\frac{\sqrt{3}}{2}\gamma \lambda_{pp}$ yields 1.4 µs (sample A), indicating that the expected order of magnitude is approximately calculated. The residual discrepancy may be related to the indirect effect of a distribution of spin–spin relaxation times. If such a distribution is not negligible but at the same time sufficiently narrow not to significantly affect the single exponential shape of the echo decay, it may be reasonable to expect some discrepancies between the relaxation rate inferred from cw measurements and the relaxation rate fitted from decay curves. In contrast with shallow donors in silicon, where the isotopic purification reduces by more than an order of magnitude the linewidth, dominated by unresolved super hyperfine interactions with ^29^Si, and significantly increases the coherence time $T_{2}$, due to the highly localized electronic wave function of substitutional N in silicon, the isotopical purification does not reveal, as expected, the same advantage in terms of $T_{2}$. A deeper analysis of $T_{2}$ requires further work in this system, possibly comparing alternative $T_{2}$ measurement techniques like the Carr–Purcell–Meiboom–Gill method. Since the focus of the present paper regards the spin–lattice relaxation mechanisms, we do not discuss this subject further.

### 3.4. Spin–Lattice Relaxation

From Figure 3 and Figure 4, the 1/$T_{1,Str}$ values show an initial steep increase, followed by a well-defined peak with comparable width for the two samples, and a further increase upon approaching room temperature. A polynomial fit of the log-log data in Figure 3 in the range [26 K, 300 K] provides an exponent of 3.4 for both samples. Notably, the peak in the spin–lattice relaxation rate falls around the temperature of 90 K, well below the onset of an appreciable shift in the position of the resonance, indicating the presence of motional averaging. The stretch exponent also shows a remarkable thermal variation: it has low values (approximately 0.5 for sample B and much lower values for sample A) at the lowest temperatures; it increases up to nearly one between the approximate temperatures of 30 K and 70 K, then upon heating further, it decreases to very low values, below 0.5; the minimum is reached at the same temperature as the peak in the relaxation rate; and finally, the exponent reaches a value close to one; however, it is lower than one at room temperature (~0.8 for sample B and 0.85 for sample A). In the range of 120 K to 150 K, the data potentially indicates a second peak towards one; since in this temperature range the intensity issues increase the error bars, this peak is considered apparent and unreliable.

Notably, the same trend for the stretched exponential recovery fit parameters is observed in both samples. This potentially indicates that the ^29^Si hyperfine interaction does not greatly contribute to the determination of the spin–lattice relaxation and that the same physical mechanism triggers these trends for both samples.

## 4. Discussion

An interpretation of the reported spin–lattice relaxation rates is tempting in terms of a peaked distribution typical for systems showing dynamical reorientation [33,34]. This interpretation implies a functional form, as follows:

(3) $$\frac{1}{T_{1}}=F\left(T\right)+D\cdot \frac{\tau_{c}}{1+\omega_{0}^{2}\tau_{c}^{2}}$$

where $F\left(T\right)$ refers to the sum of any other underlying temperature-dependent spin–lattice relaxation mechanism (direct, Raman, and Orbach mechanisms to cite the most relevant ones), D is a prefactor for the contribution due to dynamics, $\omega_{0}$ is the spectrometer working angular frequency, and $\tau_{c}$ is a temperature-dependent correlation time, generally assumed to be thermally activated [35,36], and can be calculated by the following: $\tau_{c}=\tau_{c,0}\cdot e^{-\frac{\Delta E}{k_{B}T}}$, where ΔE is the activation energy and $k_{B}$ is the Boltzmann’s constant.

However, this analysis is justified in the case of true spin–lattice relaxation times but not for the stretched case. Moreover, it would yield an unreasonably small $\tau_{c,0}$ on the order of a few units of 10^−18^ s, nearly independent of $F\left(T\right)$, and for both samples, these values are expected to be on the order of a few ps [1].

Hence, we propose a different analysis. A typical interpretation for the presence of stretched exponential relaxation relies on the existence of a distribution of single exponential relaxation rates in the physical system under investigation, as shown in EPR and other resonance techniques [37,38,39]. This distribution is wider as the stretched exponent is reduced from 1 to 0.5. The exact shape of the distribution of the spin–lattice relaxation rates cannot be extracted from the present data; however, we perform a mathematical analysis that has been reported in the literature [40,41,42] to capture more information on our physical system. We follow the analysis in Ref. [41]. The stretched exponential relaxation function in integral form can be written as a sum of single exponential rates, weighted by a probability density, as follows:

(4) $$e^{-{\left(\frac{\tau_{IR}}{T_{1,Str}}\right)}^{n}}=\int_{0}^{\infty }P\left(\frac{1}{T_{1}};\frac{1}{T_{1,Str}},n\right)e^{-\frac{\tau_{IR}}{T_{1}}}d\frac{T_{1,Str}}{T_{1}}.$$

The probability density $P\left(\frac{1}{T_{1}}\right)$, with parametric dependence on $\frac{1}{T_{1,Str}}$ and n, must be normalized by definition, $\int_{0}^{\infty }P\left(\frac{1}{T_{1}};\frac{1}{T_{1,Str}},n\right)d\frac{T_{1,Str}}{T_{1}}=1$. $P\left(\frac{1}{T_{1}};\frac{1}{T_{1,Str}},n\right)$ can be obtained by calculating the inverse Laplace transform of the stretched exponential, as shown below:

(5) $$P\left(\frac{1}{T_{1}};\frac{1}{T_{1,Str}},n\right)=\frac{1}{2\pi i}\int_{-i\infty }^{i\infty }e^{-x^{n}}e^{\frac{T_{1,Str}}{T_{1}}x}dx=\frac{1}{2\pi }\int_{-\infty }^{\infty }e^{-{\left(iu\right)}^{n}}e^{i\frac{T_{1,Str}}{T_{1}}u}du.$$

By neglecting the imaginary part, which is zero, it is possible to obtain the following:

(6) $$P\left(\frac{1}{T_{1}};\frac{1}{T_{1,Str}},n\right)=\frac{1}{\pi }\int_{0}^{\infty }e^{-u^{n}\cos\frac{n\pi }{2}}\cos\left(\frac{T_{1,Str}}{T_{1}}u-u^{n}\sin\frac{n\pi }{2}\right)du.$$

**Figure 6:**
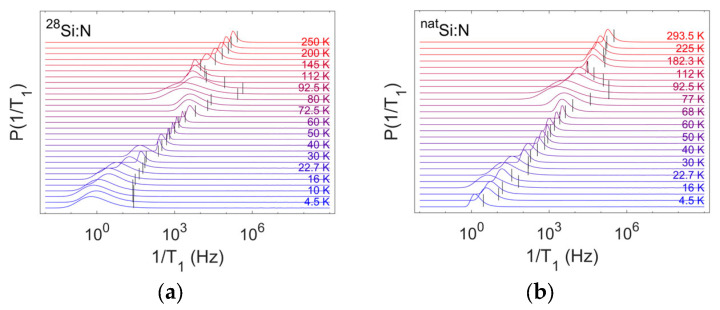
Probability distribution of single exponential spin–lattice relaxation rates. The distributions are calculated numerically by applying an inverse Laplace transform to the set of the stretched spin–lattice relaxation rates and stretch exponents for each temperature for samples A (left, panel **a**) and B (right, panel **b**). Each trace is normalized to the maximum value for better readability of the thermal variation and vertically stacked for the same purpose. Black vertical lines indicate the value of the stretched spin–lattice relaxation rate fitted from the inversion recovery curves at each temperature.

This integral can be calculated numerically starting from each set of $\left[\frac{1}{T_{1,Str}},n\right]$ values. The resulting distribution of single exponential spin–lattice relaxation rates is shown in Figure 6, where we also include an indication of the stretched rates at each temperature. As expected, the stretched rate value does not correspond to the peak of the distribution or its average. In the ranges where n approaches unity, the calculated distribution follows a Dirac function; hence, it is generally narrow and approximately centered on the fitted stretched rate. In the ranges corresponding to n≤0.5, the distribution is much broader, especially at the onset of the motional transition, and the fitted stretched rate differs significantly from the peak position of the distribution.

**Figure 7:**
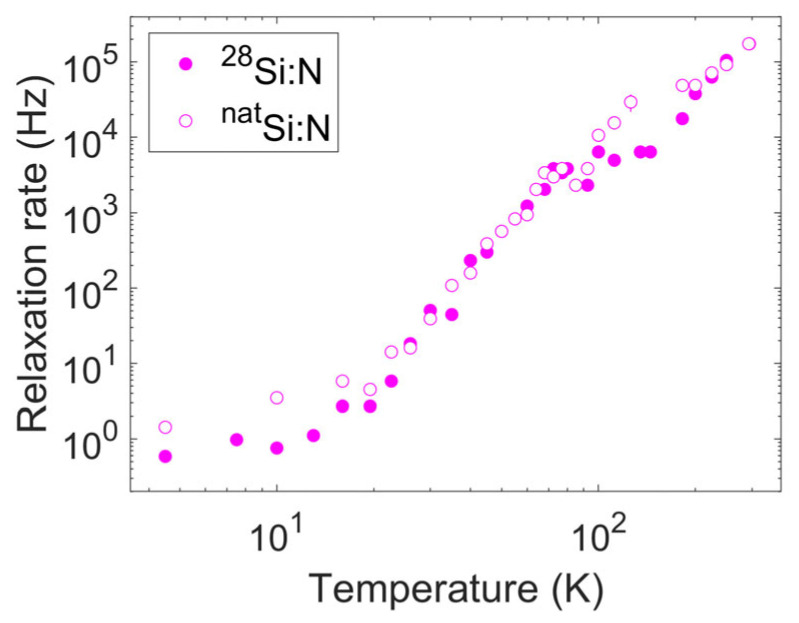
Thermal variation in the relaxation rates corresponding to the maximum of the calculated rate distributions reported in Figure 6. Data for both samples are reported (filled dots for sample A and empty dots for sample B).

As a final step in the analysis of the spin–lattice relaxation, we can now extract the maximum values of the distribution of single exponential spin–lattice relaxation rates and observe their thermal variation (Figure 7). Even though the true distribution is unknown, this quantity should provide clues on the physical mechanisms at the origin of spin–lattice relaxation in the system. As expected, for this derived quantity, the same peak due to the dynamics observed in 1/$T_{1,Str}$ at approximately 90 K almost disappeared. The analysis essentially purges the relaxation rates from the disorder effects, enabling the focus to be on the basic mechanisms of spin–lattice relaxation.

**Figure 8:**
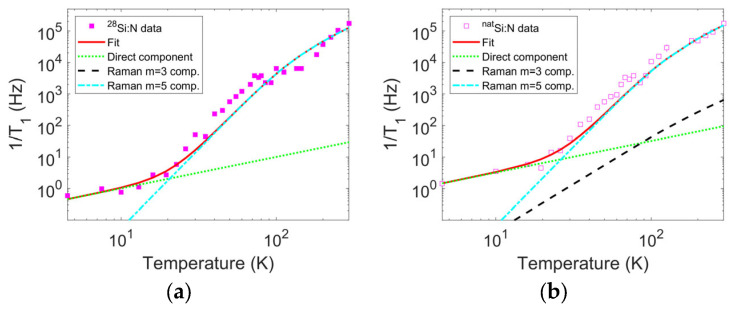
Thermal variation in the relaxation rates corresponding to the maximum of the calculated rate distributions (magenta squares) and the corresponding fit (full red trace). The fit is performed according to the model described by Equation (7), comprising direct phonon relaxation (dotted green line) and Raman relaxation (m = 3, dashed black trace; m = 5, cyan dash–dot trace). Panel (**a**) pertains to sample A and panel (**b**) pertains to sample B.

An initial tentative fit following standard fitting models [43,44,45] shows the presence of a direct phonon contribution and a Raman contribution with Raman exponent ~5, without the Orbach contribution (or any other contribution described by the same Arrhenius functional form). Due to the peculiarity of the N_Si_ system, we use an analysis already considered for other Jahn–Teller systems, which implies a trend for the spin–lattice relaxation rate expressed by [34,46,47,48]:

(7) $${\left.\frac{1}{T_{1}}\right|}_{\max\left(P\right)}=C_{1}T+C_{3}J_{2}\left(T;\Theta_{D}\right)T^{3}+C_{5}J_{4}\left(T;\Theta_{D}\right)T^{5},$$

with the following: $J_{m-1}\left(T;\Theta_{D}\right)=\int_{0}^{\frac{\Theta_{D}}{T}}x^{m}\frac{e^{x}}{{\left(e^{x}-1\right)}^{2}}dx$, where $\Theta_{D}$ is the silicon Debye temperature assumed as a constant and equal to 634 K [49]. Using this notation and performing a logarithmic fit to consider the full relaxation rate range spanning various orders of magnitude in the investigated temperature range (Figure 8), we obtain $C_{1}=0.10$ Hz/K, $C_{3}=0$ Hz/K^3^, and $C_{5}=2.1\cdot 10^{-8}$ Hz/K^5^ for sample A and $C_{1}=0.32$ Hz/K, $C_{3}=1.3\cdot 10^{-5}$ Hz/K^3^, and $C_{5}=2.6\cdot 10^{-8}$ Hz/K^5^ for sample B. We avoid declaring fitting uncertainties for these parameters on purpose since the fitted data are obtained under the assumption of an arbitrary distribution of the spin–lattice relaxation rates. The applied fitting procedure can be considered indicative of the general relaxation mechanism in the system.

According to theory, a Raman contribution with an exponent of five is linked to the second-order splitting of the (nominally equivalent) potential wells contributing to the Jahn–Teller system, while a Raman contribution with an exponent of three is related to the degeneracy of the vibrational state within a single potential well if present. Notably, the theory predicts that each Raman term is accompanied by a direct phonon like term (proportional to the temperature), and a higher direct phonon contribution prefactor is fitted in sample B due to the presence of the m=3 contribution, while the prefactors for the m=5 contributions are comparable for the two samples. In the case of sample A, the m=3 contribution is absent. The equation written above applies to the high temperature approximation, i.e., with thermal energy higher than the potential well inequivalence and the Zeeman energy, as in our case.

We propose that the origin of the distribution of values for the spin–lattice relaxation rates derives from the interplay between the active spin–lattice relaxation mechanisms in the different temperature ranges and the local environment surrounding the off-centered nitrogen atom in the substitutional position; we neglect the contribution of the centered configuration, observable only at very high temperatures [27]. Each N_Si_ is characterized by a shift towards one of the possible <111> directions for the nitrogen donor, and electron paramagnetic resonance only probes the sum of all local configurations provided by each donor in the investigated sample. In principle, the four possible configurations should be equivalent. However, we can anticipate that each donor is affected by the local configuration, i.e., by the local displacement of the nearby donors and the Si atoms close to them (at a distance related to the donor concentration); hence, the initial degeneracy is removed. By performing spin–lattice relaxation measurements on the isolated <111> line, we intrinsically select the resonance signals for the N_Si_ centers displaced along this single direction among the four possible configurations and with a ^28^Si atom as the closest Si atom (since a ^29^Si isotope would contribute to a different spectrum [1]).

**Figure 9:**
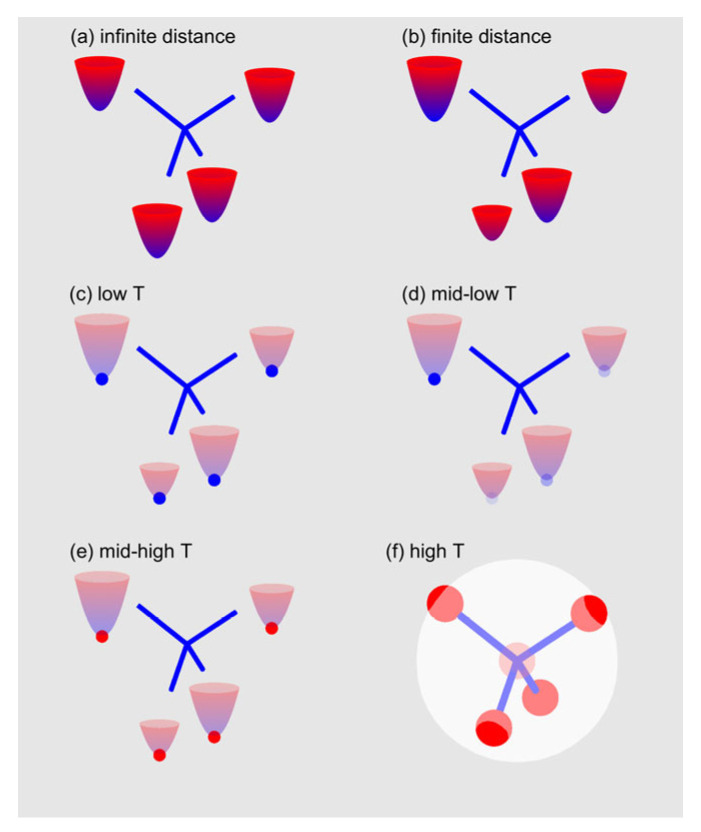
Sketch of the proposed interpretation model. (**a**) Potential wells related to the N displacements along the four <111> directions are equivalent, excluding possible mutual interactions and second-order effects;(**b**) second-order effects cause the potential wells to become inequivalent; (**c**) at low temperature, all wells are populated due to freezing from room temperature; (**d**) by heating, thermal-assisted transitions intrinsically select the local lowest energy configurations; (**e**) by further heating, the configuration jump rate increases, and all wells become populated again; and (**f**) finally, at high temperature, the system undergoes the motional transition, and only an average configuration is observed (large white sphere); in this situation, the further well related to the on-center site also begins to be populated (semitransparent central red sphere).

We propose the following interpretation:

- Each single N_Si_ center is characterized by four potential wells corresponding to the four equivalent <111> directions due to the lattice symmetry. In the case of noninteracting centers, we expect these potential wells to be identical (Figure 9a); however, mutual interaction, Si atom displacements, and second-order effects occur to remove their degeneracy (Figure 9b). Here, we neglect a 5th potential well corresponding to the on-center N configuration, reported as 73 meV above the other wells with a slightly higher energy barrier [27];
- At very low temperatures, the system is frozen in a disordered situation due to the cool-down from room temperature (Figure 9c). Each N_Si_ state corresponds to a random well that is not necessarily the true ground state, and the thermal energy does not enable the energy barrier between the different configurations to be overcome. Hence, the initial low temperature high disorder occurs;
- By heating, the thermal energy becomes (barely) sufficient to assist configurational jumps between the four different possible displacements along the <111> directions (Figure 9d). The donors can probe different local configurations, although the thermal energy is not sufficient to promote frequent jumps. Eventually, the system intrinsically selects the configuration characterized by the lowest energy on a local basis. Thus, the system is characterized by reduced disorder; hence, the spin–lattice relaxation distribution narrows, and the stretch exponent approaches one. Since this situation starts at approximately 30 K, we can estimate that the levels corresponding to the different potential wells are characterized by a difference of approximately $k_{B}T$ ~2–3 meV;
- Upon further heating, more thermal energy is available, and the thermal jumps between the different configurations become more frequent (Figure 9e). The probability for the nitrogen donors in nearby cells to be displaced along random <111> directions becomes higher, and each donor experiences a local configuration that varies frequently during the time scale of resonance experiments. The distribution of spin–lattice relaxation rates broadens again since the dynamics induce disorder in the ensemble probed by the resonance measurement;
- With additional heating, configurational jumps occur at a higher rate, and the onset of motional narrowing is observed (Figure 9f). This situation still shows a high degree of disorder, but the motional averaging reduces its effect. We can consider that in this regime, the time scale of the relaxation experiments probes the averaged configuration rather than the instantaneous configuration of the system at low temperature. Thus, the system recovers an apparent, partially ordered situation, which can explain the reduction in the spin–lattice distribution width and the increase in the stretch exponent.

Remarkably, this interpretation model seems appropriate because the peak in the stretched spin–lattice relaxation rates occurs before the onset (upon heating) of motional narrowing, which dramatically alters the spectrum.

The effect of isotopic enrichment is negligible in most of the outlined picture, but it emerges in the details of the Raman relaxation process. The presence of ^29^Si atoms in the lattice can induce a faint splitting of the vibrational ground state for each potential well and is responsible for the Raman m=3 component in the used model. Since the contribution from the m=5 Raman component dominates over the m=3 component in the entire investigated temperature range, this splitting is expected to be very small, smaller than the distance between the ground states of different potential wells. This correlates with the order of magnitude of the isotope effect on typical phonon energies in similar cases, i.e., not more than a few cm^−1^ [50,51]. The ratio of the magnitude of the two Raman components for sample B enables a very rough estimate according to [47] of ~20 µeV (Supplementary Discussion Section S5). We do not have data on the spatial characteristics of the new vibrational state considered, but it is reasonable to relate it to the slight off-axis vibrations due to the presence of a ^29^Si atom among the three Si atoms in the donor lattice cell; specifically, the Si atoms are placed along the <111> directions where the N donor did not displace its position; as mentioned above, the selected EPR transition only probes the case of ^28^Si along this displacement direction.

## 5. Conclusions

The deep donor state of nitrogen in silicon offers the possibility of using such a system for single electron tunneling devices operating at room temperature, paving the way for quantum devices operating at temperatures higher than those required by the other group V donors in silicon, despite the motional effects that impose a temperature limit. The knowledge of the basic mechanisms governing spin–spin and spin–lattice relaxation in nitrogen-doped silicon is fundamental to the design of such applications, even taking into account the fact that the greatly reduced donor concentration in any realistic tunneling device may affect the relaxation rates.

In the present article, we studied the thermal evolution of the spin–lattice relaxation rate for the SL5 center (i.e., the case of nitrogen donors at substitutional sites N_Si_) in both ^28^Si and ^nat^Si. We observe a stretched spin–lattice relaxation, potentially due to the distribution of the relaxation rates that undergo strong variations as a function of temperature from 4.5 K up to room temperature. We propose an interpretation model based on the interplay between the Jahn–Teller effect and the behavior of configurational jumps active at low temperatures; this occurs earlier than the onset of the typical motional narrowing, greatly modifies the EPR spectrum, and masks the anisotropy of the spin–Hamiltonian at high temperatures. The large depth of the nitrogen donor level yields very little difference between natural abundance and enriched systems from the point of view of spin–lattice relaxation alone. However, before releasing the requirement of a ^28^Si enrichment for realistic quantum devices based on the nitrogen-doped Si system, further investigations on the spin coherence time, possibly at lower substitutional nitrogen concentrations, should be performed. Further investigations are underway to analyze such parameters in depth using different refocusing pulse sequences.

## Data Availability

The data used in the study are available from the authors upon reasonable request.

## Supplementary Materials

The following supporting information can be downloaded at: https://www.mdpi.com/article/10.3390/nano14010021/s1, a pdf document containing Supplementary Introduction, Supplementary Discussion, and Supplementary References. The supplementary discussion is divided into: Section S1: sketch of the experimental sample preparation; Supplementary Figure S1; Section S2: SL5 center angular variation; Supplementary Figure S2; Supplementary Table S1; Supplementary Figure S3; Supplementary Table S2; Section S3: saturation curve of continuous wave signals from sample A; Supplementary Figure S4; Supplementary Figure S5: Section S4: examples of IR and ED measurements; Supplementary Section S5: estimate of the inter-well vibrational splitting. References [1,25,26,27,28,29,47,52,53,54,55] are cited in the Supplementary Materials.
