# Supplementary material for: Electron Spin–Lattice Relaxation of Substitutional Nitrogen in Silicon: The Role of Disorder and Motional Effects
Source: Nanomaterials (Basel). 2023 Dec 20;14(1):21. doi: 10.3390/nano14010021 (PMC10780458; doi:10.3390/nano14010021)
Supplement: Supplementary file 1 [file nanomaterials-14-00021-s001.zip › nanomaterials-2744419-supplementary.pdf]

Supplementary material

Supplementary introduction

In the present document we include further material (considerations, calculations, experiments, comments), in support of our article, which had to be excluded from the main article for the sake of brevity and to focus the discussion on the most important arguments.

Supplementary Discussion

Section S1. Sketch of the experimental sample preparation. The sample preparation outlined in section 2 of the main article is graphically summarized in the following scheme.

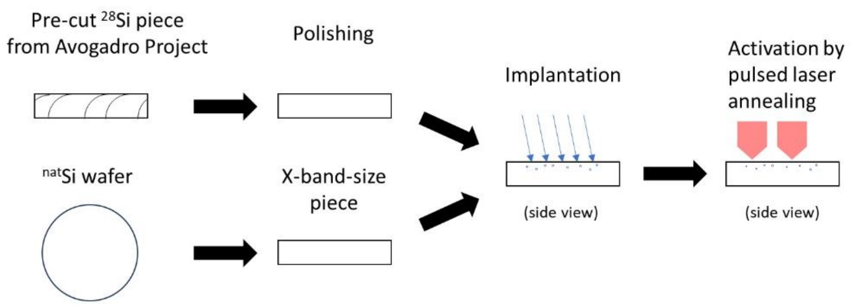

Supplementary Figure S1. Sketch of the sample preparation procedures for sample A and sample B. The expression "X-band size piece" implies the size suitable for insertion in a typical X-band resonance cavity for electron paramagnetic resonance investigations, i.e. 12 mm x 3.5 mm x 0.5 mm. The single elements of the sketch are not in scale, they are organized by focusing on the effect of the single preparation steps.

Section S2. SL5 center angular variation. The EPR investigation of both the samples evidenced the SL5 center, i.e. the N_{Si} defect, as the main contribution to the spectrum [1, 25-29]. In Supplementary Figures 2 and 3 we report the low-temperature angular variation of the cw spectra for sample A and B, respectively, together with the corresponding best fit (red lines). The fitted parameters are reported in Supplementary Tables 1 and 2. Sample A features also a different isotropic center at $g = 2.0054$, which may be ascribed to a residual contribution due to the silicon dangling bond originating from the cutting and not fully removed by the polishing treatment applied earlier than $^{14}N^{+}$ implantation. Its position is compatible with the standard $g = 2.0055$ within the experimental uncertainty. Its width is quite reduced with respect to the standard reports for the cut signals of 0.6 – 0.7 mT [52-54], a fact which may be ascribed to the effect of ^{28}Si isotopic enrichment. For the sake of completeness we add that another weak center emerges while applying higher power levels (Supplementary Discussion, section S3). It consists of a single transition, superimposed to the triply-degenerate branch in the high-field part of the SL5 center, around the value of the free electron g-factor, and may be due to a known high-temperature center, presumably created during the same pulsed laser annealing treatment applied to activate the nitrogen donors in substitutional positions [55].

The fitting of the angular variation of the cw EPR spectra was performed on the set of spectra as a whole, considering Voigtian line shapes. The same procedure did not yield satisfying results for the case of sample B, despite the lack of spurious centers. Sample B measurements were thus firstly singularly fitted with the correct number of transitions, then in a second stage the trends of the line centers were fitted to extract the Hamiltonian parameters describing the spin system. For sample B, Lorentzian line shapes were considered sufficient to fit the spectrum.

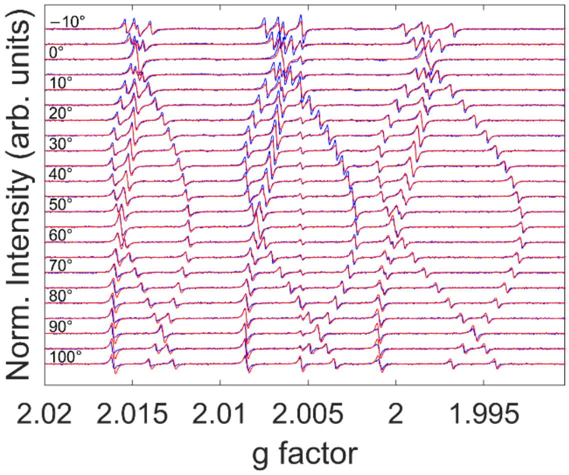

Supplementary Figure S2. Angular variation of EPR spectrum for sample A recorded at 80 K. The applied microwave power and modulation amplitude were 20 μW and 5 μT, respectively. Blue traces indicate experimental data, red traces indicate the best fit according to Hamiltonian parameters reported in Supplementary Table 1. Data are exactly the same data reported in main article Figure 1, though here the magnetic field axis is converted to a g factor scale.

Supplementary Table S1. Hamiltonian parameters obtained from the fitting of the angular variation of the EPR spectrum in sample A. σ_{p-p} and λ_{p-p} refer to the Gaussian and Lorentzian components of the peak-to-peak line width, respectively, for each center. The sample total misalignment is also calculated from two fitted parameters related to the degrees of freedom in the definition of the measurement plane. An offset in the θ angular scale is also fitted and reported in the table.

Center	Parameter	Value	Uncertainty
SL5	$g_\perp$	2.0085	1e-4
	$g_\parallel$	2.0022	1e-4
	$A_\perp$ (MHz)	36.59	0.05
	$A_\parallel$ (MHz)	46.11	0.05
	σ_{p-p} (mT)	0.5e-2	2e-3
	λ_{p-p} (mT)	3.0e-2	2e-3
cut	g	2.0054	1e-4
	σ_{p-p} (mT)	1.6e-2	4e-3
	λ_{p-p} (mT)	1.5e-2	3e-3
Weight (SL5)		4.44e-2	2e-4
Weight (cut)		8.6e-4	5e-5
$\Delta\theta$		0.4°	0.3°
Misalignment		2.5°	0.3°

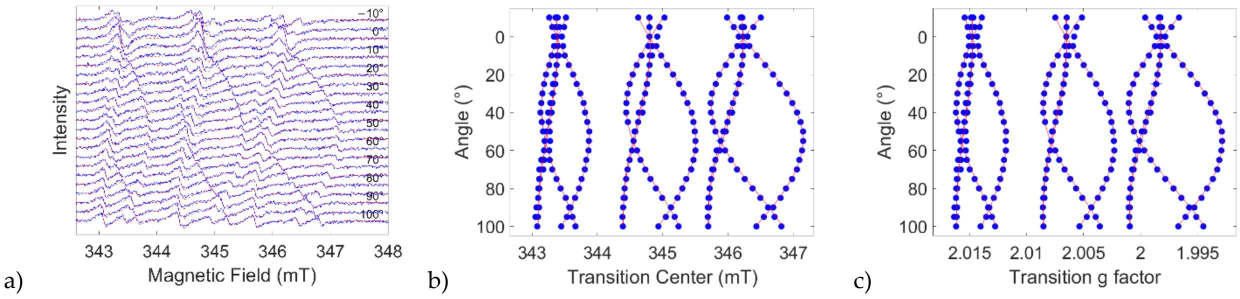

Supplementary Figure S3. Angular variation of EPR spectrum for sample B recorded at 77 K. The applied microwave power and modulation amplitude were 63 μW and 20 μT, respectively. Blue traces indicate experimental data, red traces indicate the best fit according to Hamiltonian parameters reported in Supplementary Table 2. a) Data reported as a function of the magnetic field; b) positions of the various transition centers fitted for each sample orientation; c) equivalent g factor corresponding to the centers of the various fitted transitions.

Supplementary Table S2. Hamiltonian parameters obtained from the fitting of the angular variation of the EPR spectrum in sample B. The applied microwave power and modulation amplitude were 63 µW and 20 µT, respectively. $\overline{\lambda_{p-p}}$ refers to a weighted average of the Lorentzian peak-to-peak line widths fitted for all the branches either for the low-field, central or high-field transitions, at the different orientations. Uncertainties from the line widths derive from the standard deviation of the sets of width values. The sample total misalignment is also calculated from two fitted parameters related to the degrees of freedom in the definition of the measurement plane. An offset in the θ angular scale is also fitted and reported in the table.

Center	Parameter	Value	Uncertainty
SL5	$g_\perp$	2.0085	1e-4
	$g_\parallel$	2.0023	1e-4
	$A_\perp$ (MHz)	36.56	0.05
	$A_\parallel$ (MHz)	45.92	0.07
	$\overline{\lambda_{p-p}}$ (mT) low-field	0.22	1e-2
	$\overline{\lambda_{p-p}}$ (mT) central	0.26	3e-2
	$\overline{\lambda_{p-p}}$ (mT) high-field	0.17	2e-2
$\Delta\theta$		1°	0.3°
Misalignment		1.4°	0.3°

Section S3. Saturation curve of continuous wave signals from sample A. Supplementary Figure 4 reports 80 K continuous wave X-band measurements of sample A oriented along the <111> direction as a function of applied microwave power. The sample orientation was not perfect, as observable from the slight splitting of the triply-degenerate branch in the high-field transitions. The applied modulation amplitude was 5 µT. Notably, the spectrum is dominated by the SL5 centre and the cut signal, while only at the highest microwave power levels a further signal emerges in the g-factor range 2.0023 – 2.0025, presumably due to the well-known high-temperature centre [55]. The sample did not undergo high temperature annealing, though locally the pulsed laser annealing has a similar effect. This component is essentially unobservable at the standard power levels used to probe the SL5 centre, hence it will not be discussed further here. Supplementary Figure 5 shows the saturation curve for three of the different transitions depicted in Supplementary Figure 4.

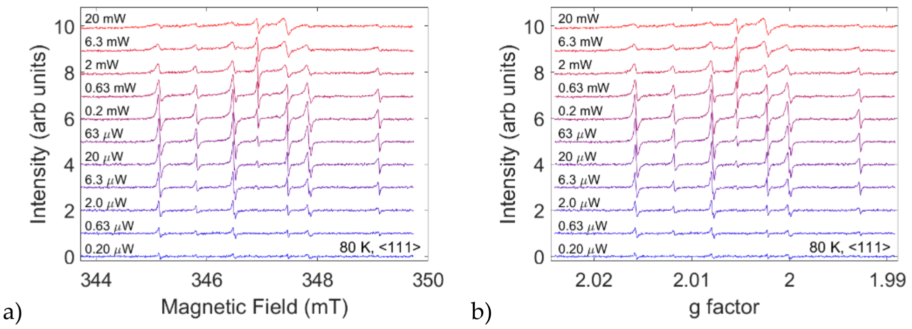

Supplementary Figure S4. EPR spectra of sample A as a function of the applied microwave power. The applied modulation amplitude was 5 µT. The six transitions due to SL5 centre and the single transition due to the cut signal are evident. A further signal emerges at high power around the value of the free electron g factor. a) Data reported as a function of the magnetic field; b) the same magnetic field span is converted to a g factor scale.

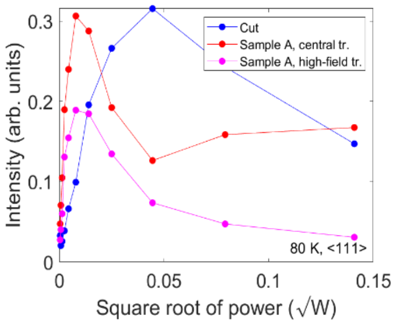

Supplementary Figure S5. saturation curves extracted from spectra in Figure S3. The saturation behaviour evidences a further component emerging at very high power at the position of the isolated branch for the central transition (red dots). This is also evident in the spectra, by comparing the intensity of the different transitions in SL5 centre.

Section S4. Examples of IR and ED measurements. In this section we report examples of inversion recovery (IR) and echo decay (ED) experiments: the first column refers to single exponential fittings of inversion recovery or saturation recovery curves; the second column to stretched exponential fittings of the same data; the third column to single exponential fitting of the echo decay data. Rows identify data taken at the same temperature (or the closest available) for ^{28}Si (first three rows) and ^{nat}Si (last three rows).

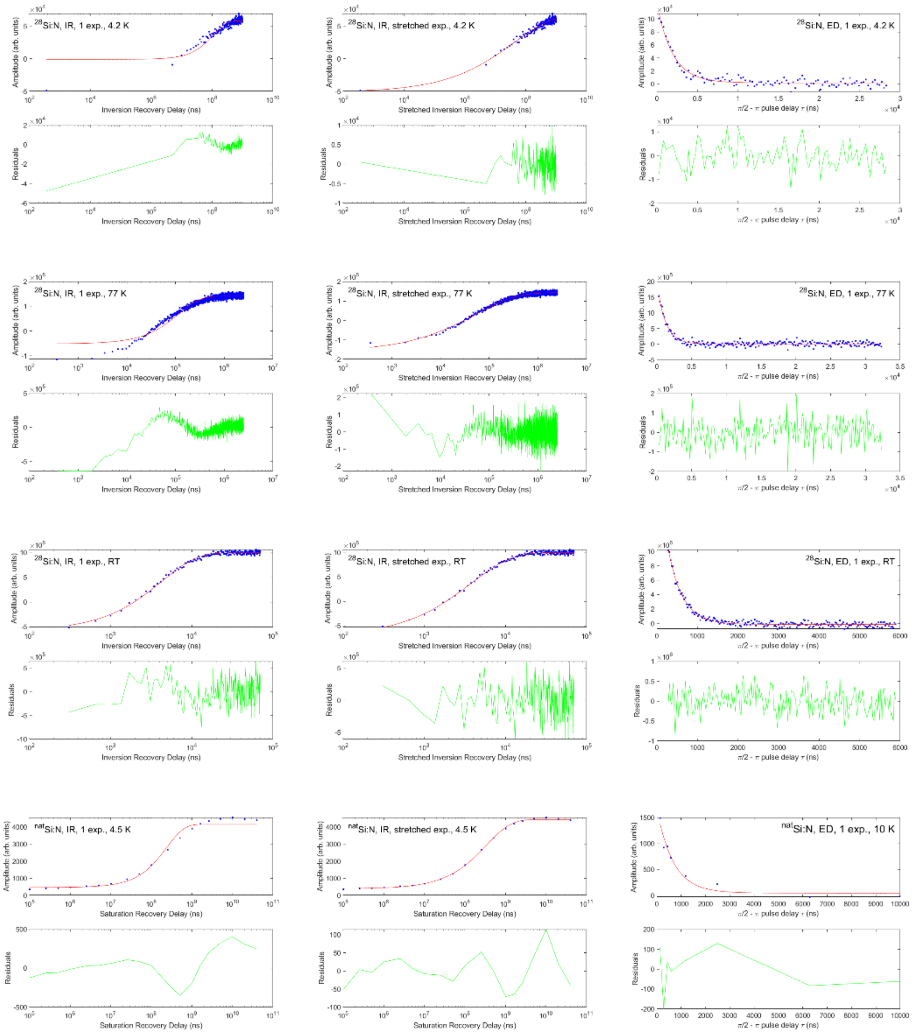

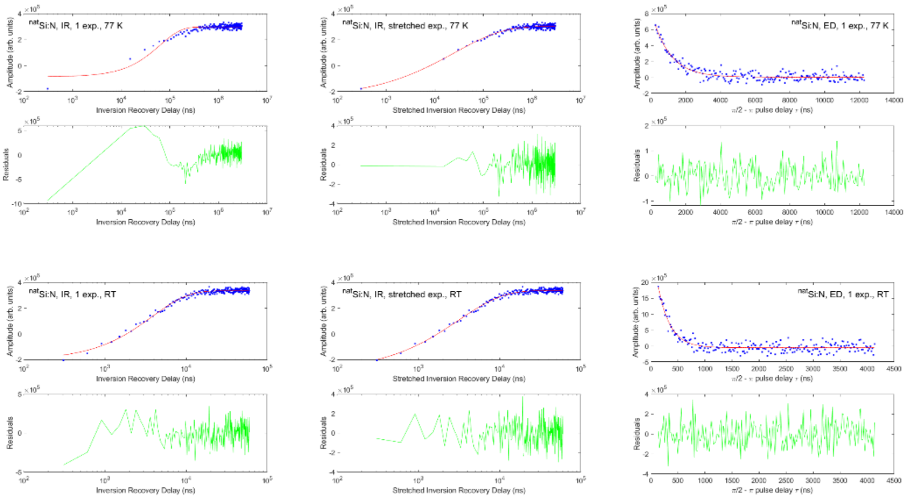

Section S5. Estimate of the inter-well vibrational splitting. By following Ref. [47], we can calculate that the ratio between the two different Raman components is:

$$\frac{\widetilde{C_3}T^3}{\widetilde{C_5}T^5} = \frac{15\Gamma^2}{8\pi^2\epsilon^2 k_B^2}\cdot\frac{T^3}{T^5}$$

where Γ represents the inter-well vibrational splitting and ϵ is a parameter related to the symmetry of the vibration. If we neglect, due to the low occurrence probability, the case of more than 1 ^{29}Si atom and that 3 positions are available for the ^{29}Si as explained in the main article, then we can assume $\epsilon = \frac{2\pi}{3}$. For the present estimate, however, to be consistent with Williams' theory, it was necessary to repeat the fitting by removing $J_{m-1}(T;\Theta_D)$ integrals in the relaxation rate expression. In this further model the fit is slightly worse, and $\widetilde{C_3}$ assumes the values $5.2\cdot10^{-15}$ Hz/K^3 and $1.2\cdot10^{-9}$ Hz/K^3 for sample A and sample B respectively, while $\widetilde{C_5}$ is $4.7\cdot10^{-7}$ Hz/K^5 and $5.6\cdot10^{-7}$ Hz/K^5.
This allows to estimate Γ as 19 μeV for sample B, and 0.043 μeV for sample A.

Supplementary References

Please, refer to the main article reference list and numbering. The same references are reported in the following for ease of reading.

1. Belli M.; Fanciulli M.; Batani D. Electron spin resonance of substitutional nitrogen in silicon. *Phys. Rev. B* **2014**, *89*, 115207. [https://doi.org/10.1103/PhysRevB.89.115207]
2. Brower K.L. Jahn-Teller-distorted nitrogen donor in laser-annealed silicon. *Phys. Rev. Lett.* **1980**, *44*, 1627. [https://doi.org/10.1103/PhysRevLett.44.1627]
3. Brower K.L. Deep-level nitrogen centers in laser-annealed ion-implanted silicon. *Phys. Rev. B* **1982**, *26*, 6040. [https://doi.org/10.1103/PhysRevB.26.6040]
4. Murakami K.; Kuribayashi H.; Masuda K. Motional effects between on-center and off-center substitutional nitrogen in silicon. *Phys. Rev. B* **1988**, *38*, 1589. [https://doi.org/10.1103/PhysRevB.38.1589]
5. Murakami K.; Masuda K.; Aoyagi Y.; Namba S. Experimental tests of non-thermal effect for pulsed-laser annealing by time-resolved reflectivity and EPR measurements. *Physica B+C* **1983**, *116*, 564. [https://doi.org/10.1016/0378-4363(83)90308-X]
6. Murakami K.; Itoh H.; Takita K.; Masuda K. Substitutional nitrogen impurities in pulsed-laser annealed silicon. *Appl. Phys. Lett.* **1984**, *45*, 176. [https://doi.org/10.1063/1.95160]
7. Williams F.I.B.; Krupka D.C.; Breen D.P. Relaxation in a Jahn-Teller System. II. *Phys. Rev.* **1969**, *179*, 255. [https://doi.org/10.1103/PhysRev.179.255]
8. Haneman D. Electron paramagnetic resonance from clean single-crystal cleavage surfaces of silicon. *Phys. Rev.* **1968**, *170*, 705. [https://doi.org/10.1103/PhysRev.170.705]
9. Lemke B.P.; Haneman D. Dangling bonds on silicon. *Phys. Rev. B* **1978**, *17*, 1893. [https://doi.org/10.1103/PhysRevB.17.1893]
10. Poindexter E.H.; Caplan P.L. Characterization of Si/SiO$_2$ interface defects by electron spin resonance. *Prog. Surf. Sci.* **1983**, *14*, 201. [https://doi.org/10.1016/0079-6816(83)90006-0]
11. Feher G. Electron spin resonance experiments on donors in silicon. I. Electronic structure of donors by the electron nuclear double resonance technique. *Phys. Rev.* **1959**, *114*, 1219. [https://doi.org/10.1103/PhysRev.114.1219]
